# Performance upgrade of a microbial explosives’ sensor strain by screening a high throughput saturation library of a transcriptional regulator

**DOI:** 10.1016/j.csbj.2023.08.017

**Published:** 2023-08-22

**Authors:** Lidor David, Etai Shpigel, Itay Levin, Shaked Moshe, Lior Zimmerman, Shilat Dadon-Simanowitz, Benjamin Shemer, Shon A. Levkovich, Liraz Larush, Shlomo Magdassi, Shimshon Belkin

**Affiliations:** a Enzymit Ltd. 3 Pinhas Sapir St., Ness Ziona 7403626, Israel; b Institute of Life Sciences, The Hebrew University of Jerusalem, Jerusalem 9190401, Israel; c George S. Wise Faculty of Life Sciences, Tel Aviv University, Tel Aviv 6997801, Israel; d Institute of Chemistry, The Hebrew University of Jerusalem, Jerusalem 9190401, Israel

**Keywords:** Bioreporters, Bioluminescence, Escherichia coli, High-throughput screen (HTS), Landmines, NGS, Precision Mutant Library, Protein engineering, Whole-cell biosensors

## Abstract

We present a methodology for a high-throughput screening (HTS) of transcription factor libraries, based on bacterial cells and GFP fluorescence. The method is demonstrated on the *Escherichia coli* LysR-type transcriptional regulator YhaJ, a key element in 2,4-dinitrotuluene (DNT) detection by bacterial explosives’ sensor strains. Enhancing the performance characteristics of the YhaJ transcription factor is essential for future standoff detection of buried landmines. However, conventional directed evolution methods for modifying YhaJ are limited in scope, due to the vast sequence space and the absence of efficient screening methods to select optimal transcription factor mutants. To overcome this limitation, we have constructed a focused saturation library of ca. 6.4 × 107 *yhaJ* variants, and have screened over 70 % of its sequence space using fluorescence-activated cell sorting (FACS). Through this screening process, we have identified YhaJ mutants exhibiting superior fluorescence responses to DNT, which were then effectively transformed into a bioluminescence-based DNT detection system. The best modified DNT reporter strain demonstrated a 7-fold lower DNT detection threshold, a 45-fold increased signal intensity, and a 40 % shorter response time compared to the parental bioreporter. The FACS-based HTS approach presented here may hold a potential for future molecular enhancement of other sensing and catalytic bioreactions.

## Graphical Abstract

**Figure:**
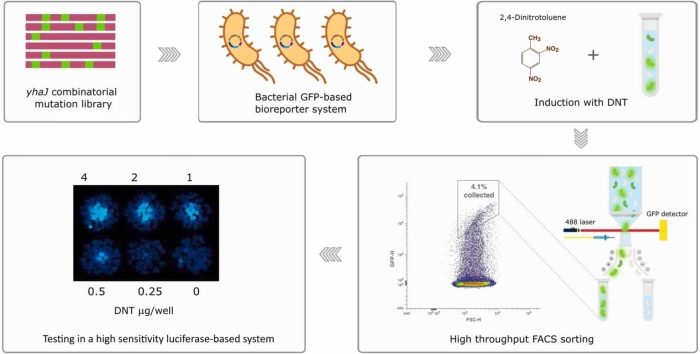

## 1 Introduction

Buried landmines and other undetonated ordnance pose a global humanitarian problem that claims numerous victims every year. Removal of landmines and other explosives is hampered by the lack of a safe and efficient method for mapping their locations. Current methods for locating explosive targets in suspected areas mostly require on-site manual search with handheld devices operated by highly trained professionals, a method that is costly, inefficient and highly dangerous. There is thus an acute need for an alternative methodology, that will allow an efficient remote detection.

One of the solutions proposed in answer to this need is the application of bacterial sensor strains, genetically engineered to generate an optical signal when sensing the presence of explosives’ molecules [22], [6]. This approach is based on the fact that traces of explosives’ vapors diffuse out of the mine’s casing and accumulate in the soil above it, generating a chemical “signature” of its presence [19]. Based on this scheme, we have previously described several *Escherichia coli* sensor strains capable of sensitively responding to the presence of TNT (2,4,6-trinitrotoluene) and DNT (2,4-dinitrotoluene) by the generation of either a fluorescent or a bioluminescent signal [29], [30], [38], [39]. We have also presented a preliminary demonstration of the standoff landmine detection capability [6]. In all of these bioreporter strains, the DNT- and TNT-inducible *E. coli yqjF* gene promoter served as the sensing element. This gene is regulated by the highly conserved transcription factor YhaJ, a member of the LysR-type family of transcriptional regulators [11], [12], [25]. A directed evolution process, based on mild error-prone PCR of both the *yqjF* gene promoter [38], [39] and the *yhaJ* gene [15], along with several chromosomal mutations in the host [29], have yielded an improved bioluminescent DNT/TNT sensor strain [15] with a DNT detection limit of ca. 40 µg/L. The reporter system in this construct was the *Photobacterium leiognathi luxCDABEG* bioluminescence gene cassette, which was found to be superior to fluorescent proteins as well as to the other microbial bioluminescence systems tested.

While practical and powerful, the low-throughput colony-based screen of a directed evolution process can deal with only a limited number of variants for each round of mutagenesis. Since YhaJ encompasses 298 amino acids, the mutational space of even a limited region of just a few positions identified to be important for function [15] is vastly larger than the space that can be screened via analysis of individual clones. To cover a mutational space that is orders of magnitude larger, there is a need for a screening technique that will significantly increase the likelihood of obtaining mutants with desired properties. In recent decades, methods for screening up to tens of millions of mutants via fluorescence-activated cell sorting (FACS) were developed. This high throughput process is widely employed to screen large antibody libraries presented on yeast, bacterial or mammalian cells [16], [18], [3], [9], as well as for screening enzymatic mutant libraries in reactions that have a fluorescent output [1], [32], [37]. There are also examples of utilization of FACS-based HTS of large libraries to improve transcription factors (TF) functionality, such as a screen for *E. coli* LacI repressor variants that respond to new inducers such as fucose and sucralose [34], or shifting the AraC response from D-arabinose to L-arabinose [33].

Here, we report on the development of a GFP-based signal system that enabled FACS screening of up to 6.4 × 10^7^ YhaJ mutant clones. We demonstrated that this system is responsive in a dose-dependent manner to DNT, enabling the screening of a large mutant library. Beneficial YhaJ mutants thus identified were then transferred to *lux*-based plasmids, in order to take advantage of the superior performance of enzymatic bioluminescent reporting compared to fluorescent proteins such as GFP. The resulting DNT reporter strains displayed significantly lower DNT detection thresholds, higher signal intensities and shorter response times compared to the parental bioreporter.

## 2 Materials and methods

### 2.1 Chemicals and reagents

DNT (2,4-dinitrotoluene) was purchased from Sigma-Aldrich (cat. no. 101397). A working stock of 12.8 g/L in ethanol was kept at room temperature. T4 DNA ligase, Exonuclease V, rSAP, and all restriction enzymes were purchased from New England Biolabs (Ipswich, MA, USA).

### 2.2 Plasmids and strains

Plasmid PBR-C55-GFPmut2-G2 (pBSO4), harboring the C55 variant of the *yqjF* gene promoter as the sensing element [29], GFPmut2 [13] as the reporting element, and the G2 variant of the *yhaJ* regulator gene [15] was employed as the basic template for the screening process. This plasmid was constructed as follows: GFPmut2 was PCR-amplified from a *yqjF*::GFP fusion [39] using primers 131_pBR_gfpmut2_F and 132_pBR_gfpmut2_R (Table S1), and the 942 bp PCR product was cloned into plasmid pBS01a (previously referred to as G2a, [15]), harboring the *Photobacterium leiognathi luxCDABEG* bioluminescence gene cassette, predigested with *EcoRI* and *NotI* using the Gibson assembly method (New England Biolabs).

To produce pBS04 with a codon-optimized version of the G2 variant, pBS04 was digested using *SalI* and *StuI* to remove the native G2 variant, and a synthesized fragment of the codon-optimized G2 was introduced using the Gibson assembly method (New England Biolabs), yielding plasmid pBS04_E.

DH5alphaZi (Expressys, Bammental, Germany) and E.cloni 10G SUPREME Electrocompetent Cells (Lucigen Corporation, Middleton, WI, USA) were used for molecular biology manipulations. *E. coli* BW25113 Δ*yhaJ* [2] was employed as the HTS host, as it was predicted that elimination of interference from wild type YhaJ would be advantageous. *E. coli* strain BW25113 Δ*ygdD*-Δ*eutE*, the superiority of which for DNT detection was previously demonstrated [29], was the host for the final bioluminescent sensors. Plasmids and host strains employed in the course of the present study are listed in Table 1, and schemes of the plasmids in Fig. S1.

**Table 1:** Bacterial strains and plasmids used in this study. Unless otherwise stated, strain BW25113 Δ*ygdD*-Δ*eutE* was employed in all the DNT induction experiments.

**Clone designation**	**Bacterial host**	**Description**	**Reference**
-	DH5α	Standard cloning strain	
ΔygdD-ΔeutE	*E. coli* BW25113 ΔygdD-ΔeutE	*ygdD* and *eutE* deleted	[29]
ΔyhaJ	*E. coli* BW25113 ΔyhaJ	*yhaJ* deleted	[2]
**Plasmid designation**	**Plasmid**	**Description**	**reference**
BS01a	pBR-C55-luxPleio-G2	*P. leiognathi luxCDABEG* driven by the *yqjF* gene promoter (version C55), regulated by YhaJ-G2.	[15](previously designated G2a)
XE/N	BS01a-XE/N	As above, with mutated YhaJ variants, with native (N) or optimized (E) codon bias.	This work
pBS04	pBR-C55-GFPmut2-G2	GFPmut2 driven by the *yqjF* gene promoter (version C55), regulated by YhaJ-G2.	This work
BS04_XE/N	pBS04_XE/N	As above, with mutated YhaJ variants, with native (N) or optimized (E) codon bias.	This work
PLD02_lib	PLD02-C55-GFPmut2-Libraray	As above, with mutated codon optimized YhaJ Library.	This work

### 2.3 Library preparation and cloning procedures

The precision six position saturation library fragments were synthesized by GenScript Biotech (Singapore).

PLD02-C55-GFPmut2-Library (pLD02) construction was conducted by amplifying the synthesized library fragments using yhaJ_sall_FW and 3prim_DbasI_Rv primers (Table S1), introducing new *HinDIII* and *BsaI* restriction sites downstream of the gene. To enable efficient cloning, the *StuI* site on pBSO4 was replaced with a *HindIII* site by digesting pBS04 with *SalI* and *StuI* and introducing a new *HindIII* site using a short DNA oligo fragment by the Gibson assembly method. The amplified library fragments were digested with *BsaI* and *SalI* and ligated by T4 ligase into the backbone of the modified pBS04, pre-digested with *SalI* and *HindIII*, in a vector to insert molar ratio of 1:16; the number of resulting transformants was ca. 1 × 10^8^. Library precision and diversity was validated by Sanger and Nanopore sequencing.

Post selection, the 6 best preforming and most sensitive YhaJ mutants (listed as clones 5,10,17,43,135,39 in Table S5) were synthesized (Twist Bioscience, South San Francisco, CA) with *E. coli* native codon usage and cloned into the backbone of pBS01a harboring the *Photobacterium leiognathi luxCDABEG* gene cassette reporter (Fig. S1) using the Gibson assembly method (New England Biolabs).

### 2.4 Exposure to DNT

For FACS-based assays, the cells were grown for 6 h in lysogeny broth (LB; Formedium, England, cat. no. LBX0202) supplemented with ampicillin (100 µg/mL) at 37 ^o^C, with shaking (200 rpm). Bacteria were washed twice in phosphate buffer saline (PBS), and diluted x1/100 in an M9 minimal medium (Formedium, UK, cat. no. MMS0101) supplemented with 2 % glucose. The cultures were induced by addition of DNT at the indicated concentrations, incubated overnight, washed twice with PBS, diluted x1/10 in PBS, and sorted by FACS. Analysis of the system’s performance and sorting of the library were carried out on a FACSAria™ III Cell Sorter (BD Biosciences, Franklin Lakes, NJ, USA).

For plate reader assays, the cells were grown in clear 96 well microtiter plates (Wuxi NEST Biotechnology, China, cat. no. 701011) for 6 h in LB, with 100 µg/mL ampicillin at 37 ^o^C with shaking (800 rpm). The cells were then washed twice with PBS, diluted x1/50 in M9/2 % glucose in black 96 well plates with a transparent bottom (Greiner, Germany, cat. no. 655090), and induced by addition of DNT at the indicated concentrations. The cells were incubated overnight at 37 ^o^C with continuous shaking in a synergy HT-T1 plate reader (Biotek, USA). Absorbance at 600 nm and fluorescence (Ex. 485 nm, Em. 530 nm) were measured every 20 min.

### 2.5 FACS screen

FACS screening was conducted using a FACSAria™ III High Sensitivity Flow Cytometer (BD Biosciences, San Jose, CA). Approximately 77 % of the main cell population was gated based on side and forward scattering (SSC-H and FSC-H, respectively). The gated main population was sorted based on GFP fluorescence (GFP-H; 100 mW laser, λex: 488 nm, with a 530 nm emission filter) as a function of forward scattering.

### 2.6 Library sequencing

Following the FACS sorting, the library plasmids were extracted after an overnight growth in LB, using a NucleoSpin Plasmid EasyPure Mini kit (Düren, Germany cat. no. 740727). Library *yhaJ* fragments were amplified using NEBNext® Ultra™ II DNA Library Prep Kit (cat. no. E7645S, New England Biolabs, Ipswich, MA, USA). DNA ends were ligated with native barcode and sequencing adapters (Native Barcoding Kit, cat. no. SQK-NBD112.24, Oxford Nanopore Technologies, UK). Nanopore sequencing was conducted on a FLO-MIN112 MiniION flow cell (Oxford Nanopore Technologies, UK) according to the manufacturer’s protocol.

### 2.7 Performance evaluation

For plate-based assays, fluorescence intensities were normalized to cell density by dividing the fluorescence value by the absorbance value at 600 nm. Following subtraction of the time zero background, induction ratios were calculated by dividing sample fluorescence at indicated DNT concentration by the fluorescence of the DNT-free control.

FACS Mean Fluorescence Intensity (MFI) was calculated by identifying and gating the major bacterial population according to forward and side scatter, and measuring the mean GFP fluorescence of the gated cells’ population.

### 2.8 Bioluminescent bioreporter responses to DNT in liquid media and on solid matrices

Bioreporter colonies harboring the relevant plasmids and carrying the beneficial *ygdD-eutE* deletion mutations [29] were grown overnight at 37 ᵒC with vigorous agitation in LB supplemented as necessary with ampicillin, kanamycin and/or chloramphenicol (100, 50 and 30 mg/L, respectively). The culture was diluted x1/100 in LB without antibiotics and regrown under the same conditions to an OD_600_ = 0.3. Bacterial aliquots (50 µl) were transferred to an opaque white 96-well plate (Greiner Bio-One), already containing 50 µl of DNT at various concentrations in 4 % ethanol. Bioluminescence was measured every 15 min at ambient temperature, using a TECAN Infinite® 200 PRO (Männedorf, Switzerland) plate reader. All experiments were conducted with internal duplicates and were repeated at least three times.

For testing the response of the bacterial bioreporters to DNT on solid matrices, they were immobilized in 3–4 mm diameter alginate beads as previously described by Shemer et al. [30], and kept at 4 ^o^C until used. The encapsulation water-based formulation consisted of 1 % alginate, 0.5 % polyacrylic acid, 3 % gelatin, 5 % polyethylene glycol 400, 1 % pullulan and 0.5 % xanthan gum. Two types of solid matrices were tested: LB agar and sand. The former was prepared by solidifying 1 mL of LB agar per well in a 48-well microtiter plate (Greiner, Bio-One), along with 10 µl of 100 % ethanol containing different amounts of DNT. The plates were left open in a chemical hood for 1 h to allow ethanol evaporation. Prior to the experiment, the encapsulated bacteria were removed from refrigeration and incubated in LB for 2 h at 30 °C with shaking (200 rpm). The beads were then drained of medium, and placed in a single layer over the LB-agar surface of the microtiter plate wells (ca. 10 beads per well). Bioluminescence was measured in a microplate reader as above, every 15 min at ambient temperature.

To evaluate the bacterial response to DNT on sand, 11-gram aliquots of beach sand were dispensed into the wells of a 6-well microtiter plate (Greiner Bio-One). Different DNT concentrations in 20 µl of 100 % ethanol were added to the center of each well, and the ethanol was allowed to evaporate for 1 h at room temperature. Encapsulated beads were spread in a single layer on the sand surface, and the plates were incubated for 10 h at room temperature in a dark chamber; images of the bioluminescent response were acquired every 20 min using a Sony α7SII camera equipped with a 28 mm lens (2–5 s exposure, f=2, ISO=5000).

Microtiter plate reader luminescence data are presented either as the instrument’s raw arbitrary relative light units (RLU), or as the difference in the intensity of the signal in the presence and absence of the inducer (ΔRLU). Detection sensitivity of the bioreporters to DNT is presented as the EC_200_ value, representing the DNT concentration at which luminescence in the presence of DNT is twice that in its absence [4], [5].

## 3 Results

### 3.1 Development of a FACS-based DNT detection and screening system

**Fig. 1:**
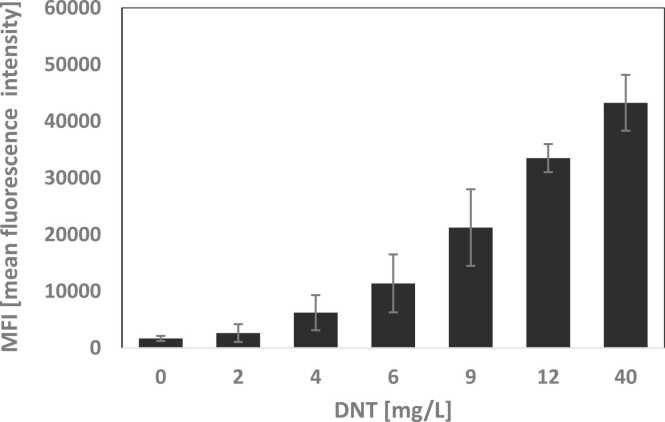
Fluorescent response to DNT of *E. coli* strain Δ*yhaJ* harboring plasmid pBS04 in a FACS assay. Fluorescence intensity is presented as mean fluorescence intensity (MFI) of the gated population.

Elad et al. [15] have previously identified several key positions in the *yhaJ* gene, mutations in which have led to an improved variant of YhaJ, the transcriptional factor regulating the induction of the *yqjF* gene promoter. To further improve DNT detection sensitivity by the latter, the sensing element in the DNT bioreporter, we have tested *yhaJ* mutants in a high throughput manner using a FACS-based screening. A GFP-based reporter system was selected, in view of a previous demonstration of an *E. coli* DNT bioreporter harboring a plasmid-borne fusion of the *yqjF* gene promoter to the GFP gene [25], [39]. The same plasmid, modified by the addition of the G2a variant of *yhaJ* [15] to generate plasmid pBS04 (Fig. S1), was used in the present study. This plasmid was introduced into an *E.coli* BW25113 strain [2] harboring a Δ*yahJ* deletion. As shown in Fig. 1, this construct responded to DNT in a dose-dependent manner, indicating that FACS sorting should be able to detect highly responsive library clones. The approximate DNT limit of detection by the pBS04 GFP-based FACS system, as may be visualized from Fig. 1, appears to be in the range of 1–2 mg/L; this is significantly less sensitive than the bioluminescent *lux*-based construct, previously shown to exhibit EC_200_ values of 0.04 ± 0.01 mg/L DNT [15]. The sensitivity disparity between the GFP- and luciferase-based systems is mostly due to the fact that the former is dependent upon the accumulation of an inert molecule, and the latter on the accumulation of active enzymes [10]. In spite of these differences, however, it is expected that any improvement in the activity of the transcription factor using GFP reporting would also benefit the activation of the lux-based system, as both reporters are driven by the same gene promoter.

### 3.2 Library design

**Fig. 2:**
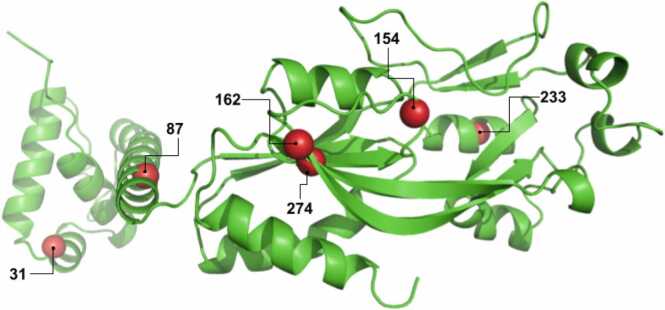
Structural view of the diversified positions targeted in the library, L31, D87, M154, I162, Q233, and A274, highlighted as red spheres. The distribution of the mutations across the protein suggests that the improved response to DNT may be a result of multiple functional domains, including both DNA interaction and ligand binding.

In a previous study [15], three iterative rounds of YhaJ engineering by error-prone PCR have led to the accumulation of six mutations in specific residues of the YhaJ protein: L31M, D87V, M154T, I162F, Q233R, and A274V (Fig. 2 and Fig. S2A). This variant demonstrated an 80-fold enhancement in DNT detection sensitivity. It has not been determined whether all of these mutations were indeed responsible for the improved activity, neither had it been demonstrated whether synergistic or additive effects were involved. Nevertheless, we have based the present study on the assumption that all of these mutations are involved in the amplified sensitivity, and on the understanding that colony-based screening of random mutagenesis clones is unlikely to identify the optimal residues or their best combination for these positions. This is due to the very large search space generated by the number of possible mutations in each location and their combinations. Given that only ca. 1,500 variants were previously examined out of the 20^300^ potential YhaJ mutants, the chance of randomly discovering the best combination of mutations for these positions was extremely low.

To find the best possible mutations, we strategically designed our library based on the 6 positions mentioned above, introducing all possible variation in these positions, yielding a library space of 20^6^ (6.4 × 10^7^) variants. Exhaustive exploration of this sequence space can be carried out by a FACS-based high-throughput system, thus facilitating the dissection of correlative effects of each position and each amino acid on DNT detection.

The native *E. coli yhaJ* gene DNA sequence contains several relatively long stretches of high GC regions, with a GC content above 70 %. To overcome the technical limitations involved in the synthesis of such regions, we have eliminated them by modifying the *yhaJ* codons. This was achieved by sampling codons from the *E. coli* codon table according to their natural frequency [28]. The resulting gene sequence had a codon distribution characterized by a low GC content that resembled that of the total codons in the *E. coli* transcriptome. The sequence of the reconstructed *yhaJ* gene (BS04_E), which served as the template for the new library is listed in Table S2. The library was subsequently cloned into pLD02_lib. Analysis of the cloned library by Nanopore massively parallel sequencing, revealed a comparably uniform diversification in all six positions, with the wild-type amino acid’s frequency approximately 10-fold higher than the rest of the mutations (25–30 % of variants, Fig. S2B, Table S6), indicating a high-quality library.

**Fig. 3:**
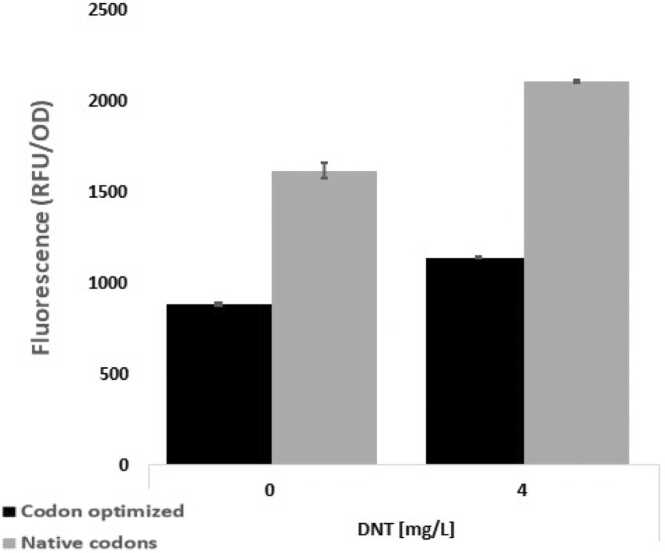
Response to DNT (4 mg/L) of the codon-optimized (black bars) and the native YhaJ template pBS04 sequence (gray bars).

Interestingly, when we tested the bioreporter containing the *yhaJ* variant with the optimized codons (pBS04_E) in comparison to the variant with the native codons (pBS04), we noticed that the strain harboring the latter responded with a much higher DNT-induced fluorescence intensity, but also with a higher background in the DNT-free control (Fig. 3, Table S3). This may be due to the fact that YhaJ belongs to the LysR-type transcriptional regulators (LTTR) family, some members of which have been shown to act both as activators and as auto-repressors [24]. It has also been specifically reported [12] that this is true for YhaJ as well. A higher expression level of pBS04_E may result in a tighter control of the *yqjF* gene promoter (C55) and a lower background expression. However, Palevsky et al. [25] have reported that a *yhaJ* null mutation is nonresponsive to DNT, indicating that YhaJ is an activator. The lower signal and lower background may alternatively be due to a destabilizing effect of pBS04_E on the mRNA, resulting in reduced protein expression [36]. It is also possible that altered translation speed due to a shift in codon composition causes protein unfolding, leading to diminished binding to DNA and subsequently to a lower response to DNT.

### 3.3 FACS selection and clone characterization

**Fig. 4:**
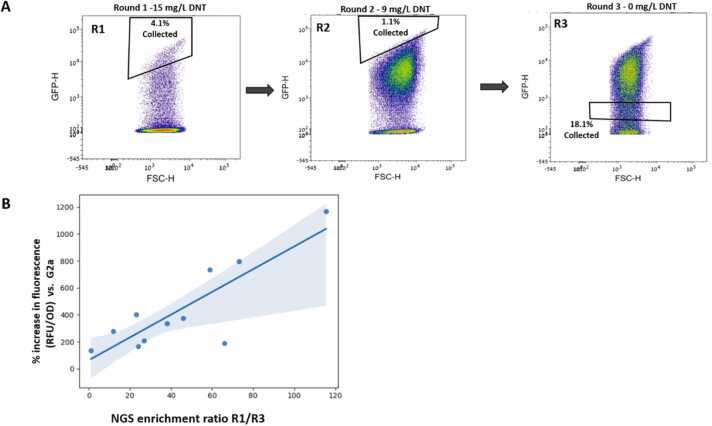
(A) FACS density plots of the *E. coli* culture harboring the pLD02 library, induced by 15 mg/L DNT (R1), 9 mg/L DNT (R2) and without DNT (R3). DNT induction levels and sorting gates are indicated. (B) Correlation between the next generation sequencing (NGS) enrichment ratio and single clone fluorescence intensity levels: out of the 17 single clones assayed, 11 were identified in the NGS sequenced population. Their enrichment ratio was calculated by comparing their frequency in the library pre- and post-sorting. The Pearson correlation coefficient (calculated by R-squared) was 0.71.

The library described above was subjected to three FACS selection rounds, the first of which was conducted following the exposure of the library to 15 mg/L DNT. In total, 9 × 10^7^ cells were scanned, sampling approximately 75 % of the library space. The top 4.1 % (ca. 3.7 ×10^6^ clones) of the highest fluorescence events were selected for the second round, in which they were induced by 9 mg/L DNT, yielding a selection of the top 1.1 % events. A large fraction of the population selected in the second round exhibited a strong fluorescence signal even in the absence of DNT. To eliminate such false positive mutants, a final negative selection round was conducted. The library was grown without DNT, and clones with minimal fluorescence (ca. 18.1 % of the total population) were sorted (Fig. 4 and Fig. S3B). A summary of the sorting statistics is presented in Table 2. Analysis of Nanopore sequencing long sequence reads performed on DNA extracted from the cells selected in rounds 1 and 3 indicated a defined clone population that was significantly enriched (Fig. 4b). The 150 best performing sequences are listed in Table S3.

**Table 2:** Sorting statistics.

Round	DNT concentration (mg/L)	Events scanned	Cells collected	Events collected out of scanned events (%)
R1	15	9 × 10^7^	3,680,000	4.1
R2	9	8.6 × 10^6^	500,000	1.1
R3	0	5 × 10^6^	712,000	18.1

**Fig. 5:**
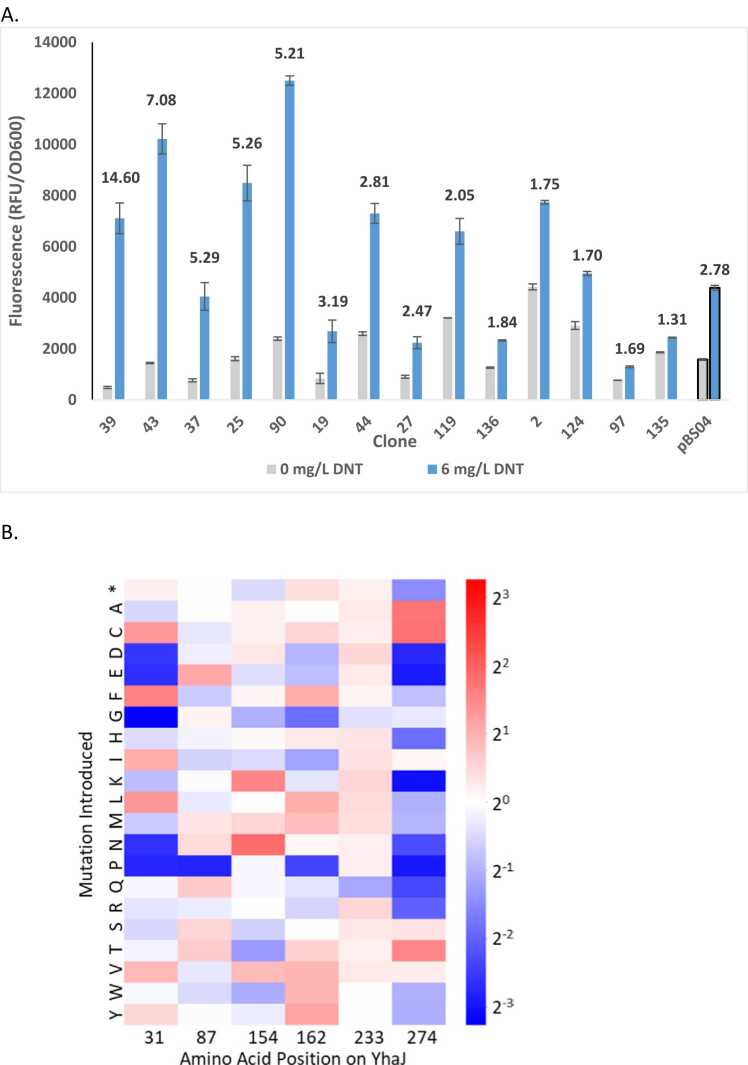
(A) Fluorescent response of selected mutants to 0 and 6 mg/L DNT in a plate reader assay. (B) Mutation enrichment heat map. The enrichment of each position and each mutation was calculated independently by computing the log2-ratio of each mutation/position combination in the library, before and after the three sorting rounds. Gradient color represents a log2-fold enrichment over the original template amino acid position.

The selected clones were plated on LB agar, and individual clones were tested for their fluorescent response to DNT (6 mg/L) both by FACS and by a 96-well plate reader. Based on the good correlation between the two assays (Figs. S4A and S4B), further testing was carried out in a 96-well plate reader. In total, 157 clones were tested, approximately 34 % of which displayed a low fluorescence background and a robust response to DNT compared to pBS04 (Fig. 5A, Table S4), indicating that approximately a third of the sorted clones underwent the desired positive and negative selections. Analysis of the mutations of the best 17 clones showed that several positions converged to a smaller set of mutations. Position 274 (valine in G2a and alanine in the wild-type variant) converged strongly to threonine, indicating that this mutation plays a key role in the bioreporter’s sensitivity and signal intensity. Another notable position is T154N or T154K (threonine in the G2a variant and methionine in the wild-type variant). Lastly, position 87 shows a strong preference towards charged or polar amino acids, suggesting that this mutation may play a role in the interaction with the DNA (Fig. 5B).

### 3.4 Bioluminescent analysis of the best performers

**Fig. 6:**
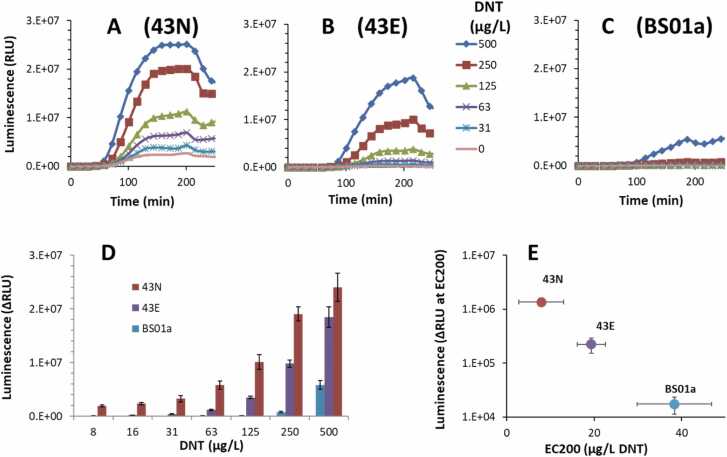
Bioluminescent response to DNT of variant strains 43E (optimized codon usage) and 43 N (native codon usage) strains, compared to the original BS01a. All three plasmids harbored the C55 variant of the *yqjF* gene promoter as the sensing element and *P. leiognathi’* s *luxCDABEG* gene cassette as the reporter element, and were hosted in *E. coli* BW25113 Δ*ygdD*-Δ*eutE* (28 ^o^C, 96-well plate format). (A-C) Signal development over time in the presence of different DNT concentrations, by clones 43 N, 43E and BS01a, respectively. (D) Bioluminescent response to DNT (maximal luminescence in the presence of DNT minus that in its absence (ΔRLU), over a 6 h exposure) of variant strains 43E (optimized codon usage) and 43 N (native codon usage), compared to the original BS01a. (E), Maximal ΔRLU over 6 h at a DNT concentration equal to EC_200_ (DNT concentration at which luminescence was twice that of the DNT-free control) plotted against the EC_200_ values.

To further test and characterize the selected mutants, the most promising 11 clones were sub cloned into the highly sensitive pBS01a system, where YhaJ controls transcription of the *luxCDABEG* cassette driven by the *yqjF* gene promoter. Six of these variants were also synthesized with the native codon usage and similarly cloned (Table S5). All clones were tested for the response to a series of DNT concentrations in liquid medium, and their detection sensitivity (as EC_200_), signal intensity (luminescence at the EC_200_ DNT concentration) and response time (time required to reach a luminescence intensity two-fold higher than the control) were calculated (Table 3). Out of the clones tested, strain 43 appears to stand out, both in the native (43N) and optimized (43E) codon usage variants. While several other clones, such as 17E and 17N, exhibited a higher signal intensity, they were inferior to both 43 versions in terms of either detection sensitivity and/or response times. When the clones with the native codon bias and the optimized ones were compared, in most cases the native codon bias exhibited a higher signal than the codon optimized one (Fig. 6); these “N” strains, however, were also characterized by a significantly higher background (uninduced) luminescence (not shown). Also evident from Table 3 is the faster response of the 43E and 43N variants: 87 min and 72 min, respectively, compared to 118 min by strain BS01a.

**Table 3:** Response to DNT by selected bioluminescent YhaJ variants, hosted in *E. coli* BW25113 Δ*ygdD*-Δ*eutE*, with *P. leiognathi luxCDABEG* driven by the *yqjF* gene promoter (version C55) at 28 ^o^C.

YhaJ clone	EC_200_* (μg/L DNT)	Luminescence at EC_200_ (ΔRLU)**	Time to ratio = 2 (min)
**5E**	53	8,000	100
**5N**	41	5,000	100
**10E**	41	71,000	100
**10N**	32	512,000	114
**17E**	161	4,691,000	474
**17N**	262	6,269,000	588
**119E**	65	51,000	119
**135E**	113	20,000	108
**135N**	122	413,000	198
**136E**	59	4,000	108
**43N**	10	1,707,000	72
**43E**	23	123,000	87
**BS01a**	70	37,000	118

* DNT concentration at which luminescence intensity is two-fold higher than that of the DNT-free control.

** Luminescence in the presence of DNT minus that of the DNT-free control (ΔRLU)

DNT detection performance of strains 43E and 43N is shown in Fig. 6, in comparison with strain BS01a. Panels A-C display the raw luminescence of the three strains in response to several DNT concentrations, and in panel D the maximal responses of all three strains as a function of DNT concentration are plotted. These curves were used to calculate the EC_200_ values; in panel E, these are plotted against the luminescence intensity of each strain at that DNT concentration. The data in Fig. 6E clearly demonstrate the significantly higher luminescence intensity of the two modified YhaJ strains, with that of variant 43N higher by one order of magnitude than that of 43E, and 45-fold higher than BS01a. Detection threshold, presented here as the EC_200_ value, is lower in both modified strains by ca. 3 and 7-fold (in 43E and 43N, respectively; Table 3).

### 3.5 Bioreporter responses to DNT on solid matrices

**Fig. 7:**
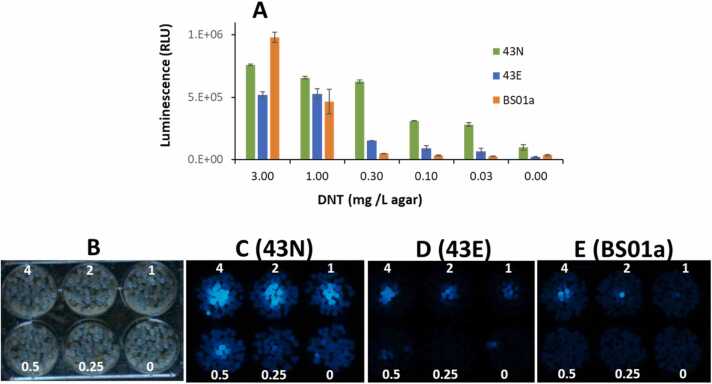
Bioluminescent response to different DNT concentrations on LB agar (A) and sand (B-D), by alginate-encapsulated variant strains 43E (optimized codon usage) and 43 N (native codon usage), compared with the original BS01a strain. All three plasmids harbored the C55 variant of the *yqjF* gene promoter as the sensing element and *P. leiognathi’* s *luxCDABEG* gene cassette as the reporter element, and were hosted in *E. coli* BW25113 Δ*ygdD*-Δ*eutE.* Values in panel A represent maximal plate reader luminescence values over a 6 h exposure on solid agar in a 48-well plate format. In panels B-E, the 6-well plates were imaged in the light (B) or in a dark chamber (C-E) after a 4 h room temperature exposure by a Sony α7SII camera equipped with a 28 mm lens (2–5 s exposure, f=2, ISO=5000). The in-image values denote the amount of DNT (µg per well) in each of the wells.

The performance of the 43E and 43N bioreporter variants in response to DNT was also assessed on two solid matrices: LB agar and sand, and compared to the BS01a control. For this purpose, they were encapsulated in alginate beads, ca. 3 mm in diameter, as described in Materials and methods. The beads were spread in a single layer over the tested surface, and luminescence was monitored over time in a microtiter plate reader (LB agar) or by a Sony α7s camera (sand). Luminescence intensity as a function of DNT concentration is shown in Fig. 7A; the advantages of strains 43E and 43N over BS01 are particularly obvious at the lower DNT concentrations. Also clear is the stronger luminescence emitted by strain 43N, evident in the DNT-free control as well.

Fig. 7B is a visible light image of the experimental setup of the sand exposure experiment in a 6-well microtiter plate, with a notation of the amounts of DNT in each of the wells. Figs. 7C, 7D and 7E are images acquired in the dark, 4 h after layering the alginate-immobilized strains 43N, 43E and BS01, respectively. Strain 43N stands out in both its stronger luminescent signal as well as in its enhanced detection sensitivity: it is the only bioreporter with a clear signal over 0.5 mg DNT/Kg sand.

### 3.6 Machine learning-assisted structural analysis

Previous reports have strongly suggested that the YhaJ transcription factor does not respond directly to DNT, but rather to 2,4,5-trihydroxtoluene (THT), an intermediate in the *E. coli* DNT degradation pathway [31]. To better understand the impact of each of the mutations on the transcription factor's affinity to the THT ligand, we computationally docked THT using DiffDock [14], a novel diffusion-based generative neural network for protein docking, to an AlphaFold2 [21] model of YhaJ downloaded from Uniprot [35].

**Fig. 8:**
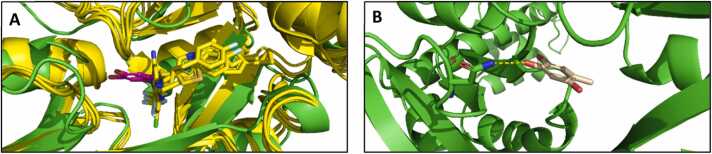
Structural analysis of the potential binding site. (A) Protein data bank structures of YhaJ homologs are superimposed together, complexed with bound ligands that were present in the crystal structures (PDB IDs 6Z5K, 7NTBW, 6Q7W, in yellow). (B) YhaJ (Uniprot P67660) AlphaFold2 model in green, and the THT DiffDock prediction in pink.

Examination of the 5 highest-scoring complexes indicated that THT occupies a conserved pocket found in multiple homologs of the LysR fold, as depicted in Fig. 8, where several solved structures of the LysR fold homologs, obtained from the Protein Data Bank [8], are superimposed [17], [27], [40]. Furthermore, out of the six mutated library positions, only one (154) is located in the vicinity of the pocket, while positions 31 and 87 are part of the helix turn helix DNA binding domain, and positions 162 and 274 are located on a protein core facing beta sheets that do not come in contact with the putative ligand binding site (Fig. 8). The impact of the distant positions on the overall sensitivity and specificity of the system may be attributed, in the case of positions 31 and 87, to an allosteric or another type of long-range interaction with the DNA. The effect induced by mutations in positions 162 and 274 can result in changes in the volume of the protein’s hydrophobic core, a parameter that has been demonstrated to induce conformational changes resulting in binding affinity modulation [7]. Position 233 is located on a solvent-exposed region, and has a glutamine residue in its native sequence. Most enriched mutations, including variants that were improved in previous studies [15], incorporated charged or polar amino acids in that position, suggesting that its role involves interactions with the solvent. Lastly, position 154 (methionine in the native variant and threonine in the G2a directed-evolution derived variant), is the only residue in proximity with the putative binding pocket of THT. The amino acid asparagine is the most enriched in this position, but some variants incorporate alanine or valine. We have employed RosettaDesign [23] to introduce the T154N mutation to the AlphaFold2 model of YhaJ. Interestingly, all the models converged to the same asparagine rotamer that positions the carboxamide group 3.7 Å away from one of the hydroxyl groups of THT (Fig. 8). Overall, this machine learning-assisted analysis raises potential hypotheses regarding the control of YhaJ’s specificity and sensitivity to its putative substrate. The mechanism induced by the mutations studied seems to mostly include long range interactions that do not involve the binding pocket. Further experimental studies are required to validate in greater detail the role of each of these positions, as well as other key loci, and elucidate the direct mechanism of the ligand specificity and sensitivity.

## 4 Discussion

We described herein a fluorescence-based HTS methodology for the identification of transcription factor modifications that enhance the DNT detection capabilities of live *E. coli* sensor cells. Coupling a GFP-based system with a focused variation library and a highly efficient FACS analysis has enabled us to screen over 75 % of the library's mutational space (6.5 ×10^7^ variants), leading to a significantly higher performance of the YhaJ transcription factor modifications. The clear DNT dose-response evident from the FACS-based assays suggest that a FACS screen of bacterial cells is indeed a robust method for directed evolution of transcription factors such as YhaJ.

The approach presented in this study has several advantages: (a) A large number of mutations, up to tens of millions per library, may be screened. (b) The procedure may be fine-tuned to the desired mutants’ characteristics. For example, negative selection in the last sorting round was used to reduce a significant number of false positive clones. Depending on mutation position and composition, it is likely that in some cases more than one or two rounds of negative selection may be required. (c) A focused library combined with a FACS-based HTS can identify mutations that have a synergistic effect on the transcription factor’s activity, which would be difficult or impossible to detect by conventional methods, especially where individual mutations may be neutral or even deleterious. Conventional approaches based on a randomly mutated library, followed by a screen of a handful of clones by plate-based analysis, cannot identify the desired combinations, due the low likelihood of generating and identifying synergistic combinations of mutations. Furthermore, an iterative selection process that accumulates mutations over several library generations will not identify individual non-beneficial mutations.

For the FACS screening stage, the original *yhaJ* gene as well as the library variants were synthesized using codons that were sampled randomly from the *E. coli* codon usage frequency table. Interestingly, although the response ratios and limits of detection by mutant 43 were similar for both codon usage schemes, in the variants that were synthesized using randomly sampled codons (codon-optimized), the signal was much lower than the comparable clones with *E. coli*’s native DNA sequence for both DNT and DNT-free conditions. Since some of the native *yhaJ* codons are rare, a possible explanation for this phenomenon might be a more efficient translation of the codon optimized variant, resulting in higher concentration of YhaJ in the cytoplasm, and consequently a tighter control of the *lux* operon in both basal expression and DNT induction scenarios. Indeed, several recent studies [20], [26], [36] have outlined the effect of codon usage on the overall expression rate and functions of translated proteins. The case presented here serves as another testament to that effect.

Structural analysis of the YhaJ protein and its THT binding pocket, using the neural networks Alphafold2 and DiffDock, shows that the mutations are located in several different areas of the transcription factor. Of the six targeted positions, only one mutation (position 154) is in an area with proximity to the THT binding pocket, while the other positions could potentially be involved with allosteric conformational changes and DNA binding. This distribution suggests that the true potential of engineering a more sensitive, stronger, and faster response to DNT is far from trivial; it reinforces the notion that transcription factor binding and dissociation are synergistic, concerted events that involve the rearrangement of large regions of the protein. Out of six mutated positions, only three showed significant preference to specific mutations, suggesting they are indeed involved in the regulatory function. It thus seems plausible that the search for optimal YhaJ performance is far from exhausted, and future libraries may discover additional beneficial positions.

The saturation library generated in this study has been designed to incorporate all amino acid combinations in a small number of positions, proposed in a previous study to have some effect on YhaJ activity. This approach can be improved upon in future studies by narrowing down the number of allowed amino acids per position, thereby allowing the incorporation of more variable positions without changing the library size and affecting screening efficiency. Such a future library may be designed by incorporating prior knowledge about other members of the same protein fold, including in the design amino acids observed more frequently than would be expected under the null hypothesis in other homologs. Similarly, amino acids appearing at lower frequencies may be excluded.

Finally, as previously mentioned, the YhaJ transcription factor may bind one or more metabolic by-products of DNT degradation. This may lead to a delayed response of the signal and could also lead to false positive responses. To address this, future libraries may focus on redesigning a variant of the transcription factor with high affinity to DNT itself, which would facilitate a more rapid response, as well as contribute to an improved detection sensitivity.

## Authors’ statement

All data that are not presented in the manuscript and in its supplementary materials will be made readily available upon a reasonable request.

## Conflict of Interest

The authors declare no conflict of interest related to our submitted manuscript **“Performance upgrade of a microbial explosives’ sensor strain by screening a high throughput saturation library of a transcriptional regulator”.**
