# Supplementary material for: Performance upgrade of a microbial explosives’ sensor strain by screening a high throughput saturation library of a transcriptional regulator
Source: Comput Struct Biotechnol J. 2023 Aug 22;21:4252–60. doi: 10.1016/j.csbj.2023.08.017 (PMC10493890; doi:10.1016/j.csbj.2023.08.017)
Supplement: Supplementary file 1 — Supplementary material [file mmc1.docx]

**Supplementary materials**

**Performance upgrade of a microbial DNT bioreporter by a high throughput saturation library of a transcriptional regulator**

Lidor David^1^*, Etai Shpigel^2^*, Itay Levin^1^*, Shaked Moshe^2^, Lior Zimmerman^1^, Shilat Dadon-Simanowitz^2^, Benjamin Shemer^2^, Shon Levkovich^3^, Liraz Larush^4^, Shlomo Magdassi^4^ and Shimshon Belkin^1^**

^1^Enzymit Ltd. 3 Pinhas Sapir St. Ness Ziona, 7403626, Israel

^2^Institute of Life Sciences, The Hebrew University of Jerusalem, Jerusalem 9190401, Israel

^3^The George S. Wise Faculty of Life Sciences, Tel Aviv University, Tel Aviv 6997801

^4^Institute of Chemistry, The Hebrew University of Jerusalem, Jerusalem 9190401, Israel

**Figure S1**


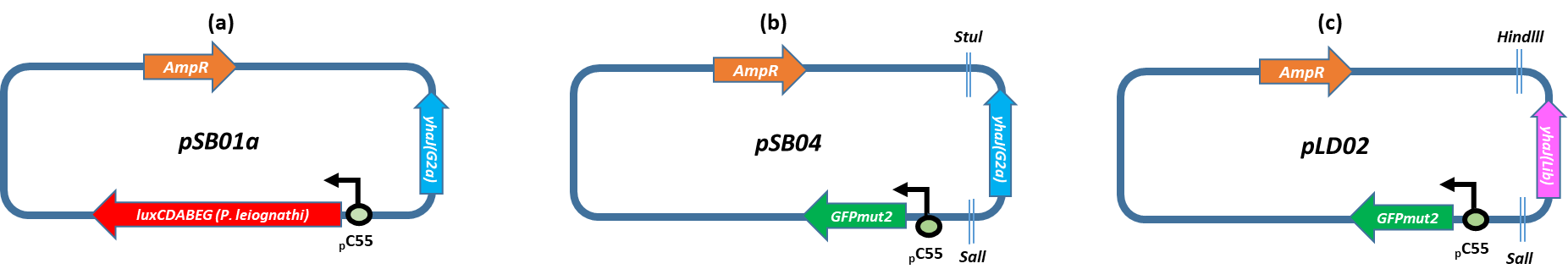


**Figure S1.** Schemes of plasmids used in this study**.** (A) plasmid pBS01a, harboring the previously modified *yhaJ* gene (G2a) with its native promoter (light blue), the *Photobacterium leiognathi luxCDABE* gene cassette as the reporter element (red), and the *yqjf* gene promoter (version C55, Yagur-Kroll et al 2015) controlling the reporter gene (light green). (B) plasmid pBS04, similar to pBS01a, but with the GFPmut2 gene as the reporter (green). (C) As pBS04, but with the *HindIII* restriction site replacing *StuI*. This plasmid served as the skeleton for the construction of the screening library(pink). In all three plasmids, the sensing element driving reporter expression was the C55 variant of the *yqjF* gene promoter.

Figure S2

A

**
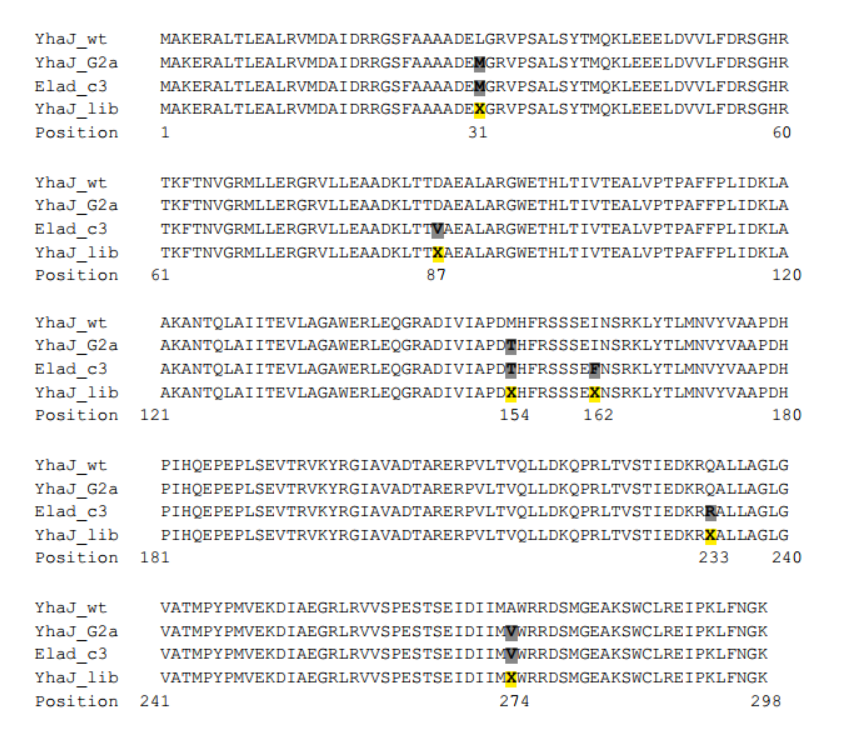
**

B


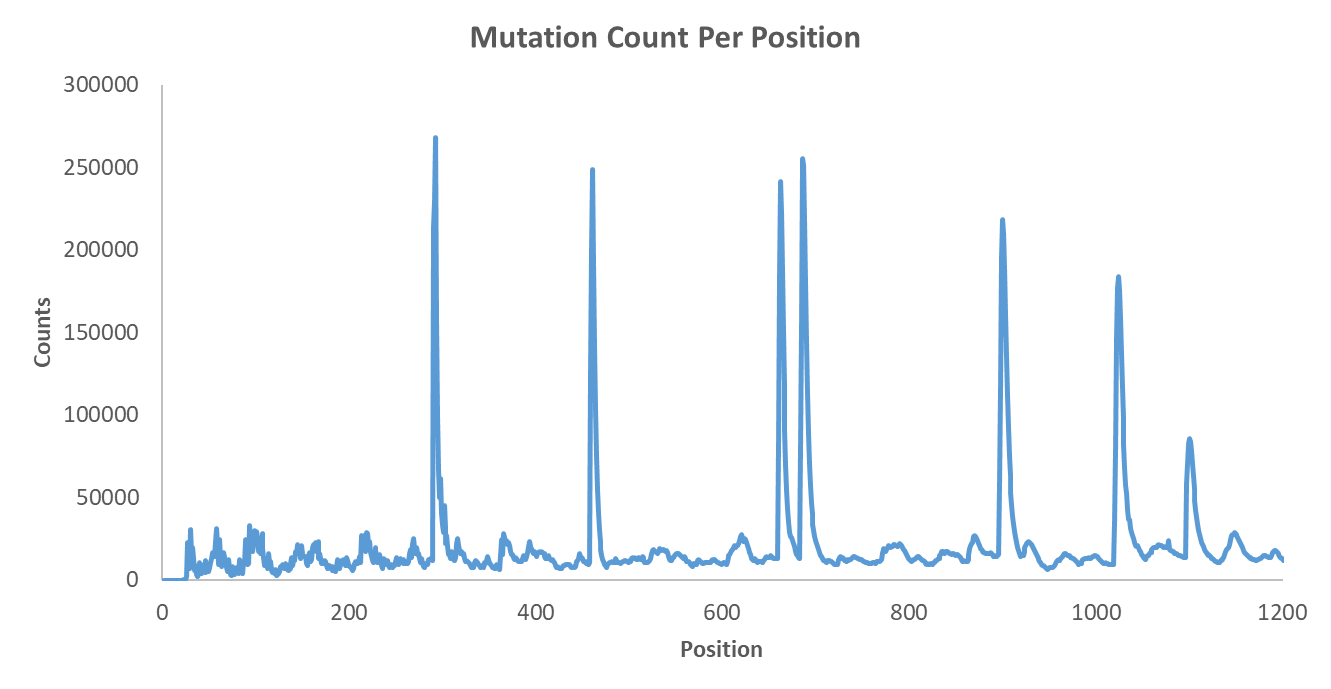


**Figure S2.** Alignment of the amino acid sequence in the different YhaJ variants. Positions marked by an X in the library sequence were diversified to all possible 20 amino acids (theoretical library space: 20^6^=6.4⨉10^7^) (A). Library Nanopore sequencing: frequency of positions diverted from yahJ Elad_c3 variant. The small right hand peak represents a “silent” 1bp mutation introduced post library preparation to eliminate a *BsaI* site (B).

**Figure S3.**

**A**


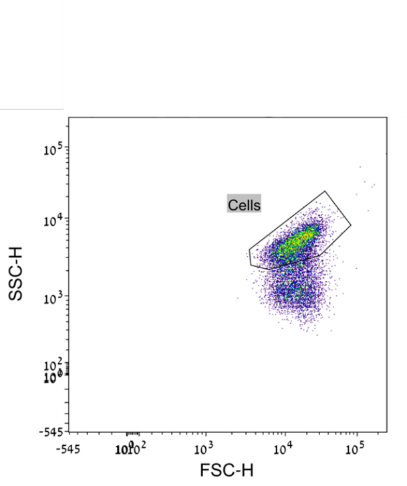


**B Round 1**

**0 mg/L 15 mg/L**


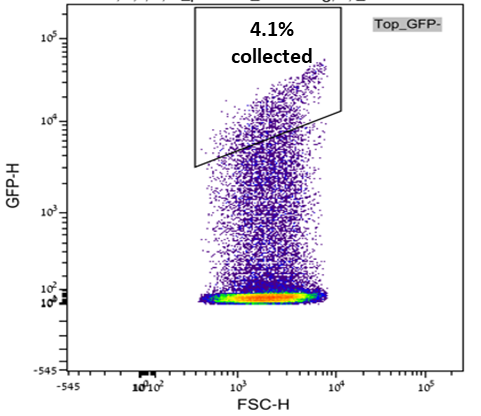

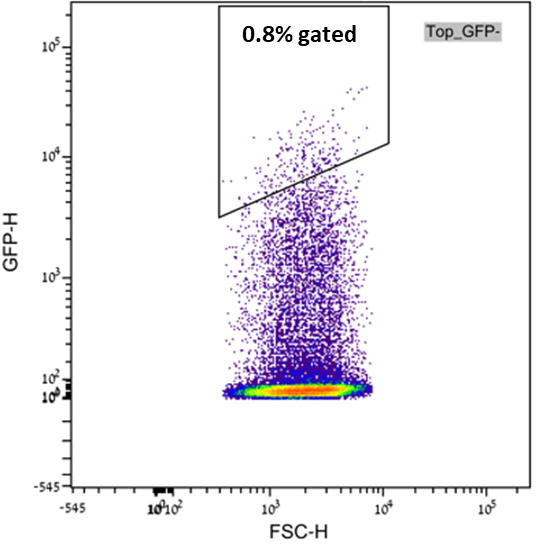


**Round 2**

**0 mg/L 9 mg/L**


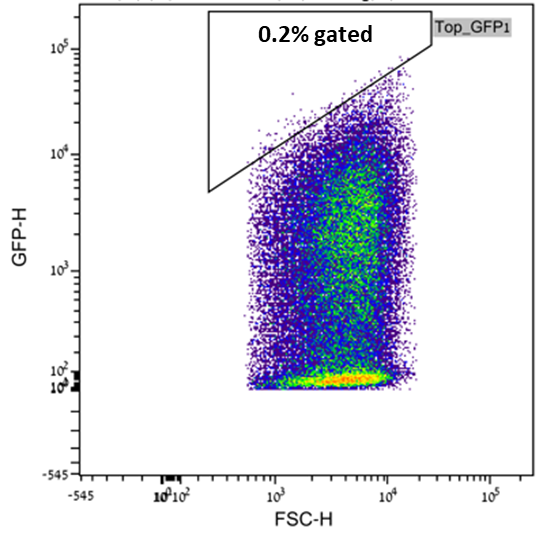

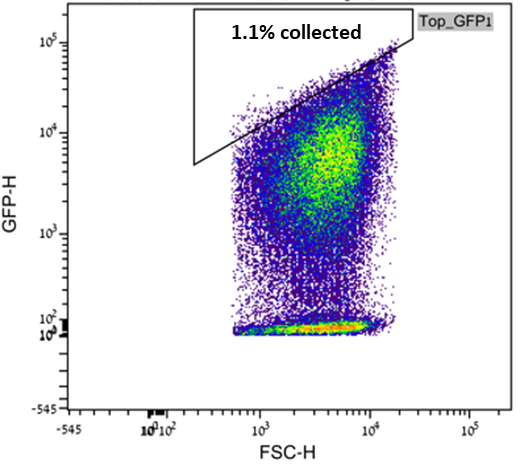


**Figure S3.** Typical main population gating strategy. Main population of cells was selected by Side scatter - SSC-H and Forward scatter – FSC-H in order to examine the cells in the best physiological condition (A). Three selection rounds gating strategy. Left panels indicate population fluorescence without DNT induction and right panels with indicated concentration of DNT, cells were collected post induction with 15, 9, 0 mg/L DNT for rounds 1,2 and 3 respectively (B).

**Round 3**

**0 mg/L 9 mg/L**


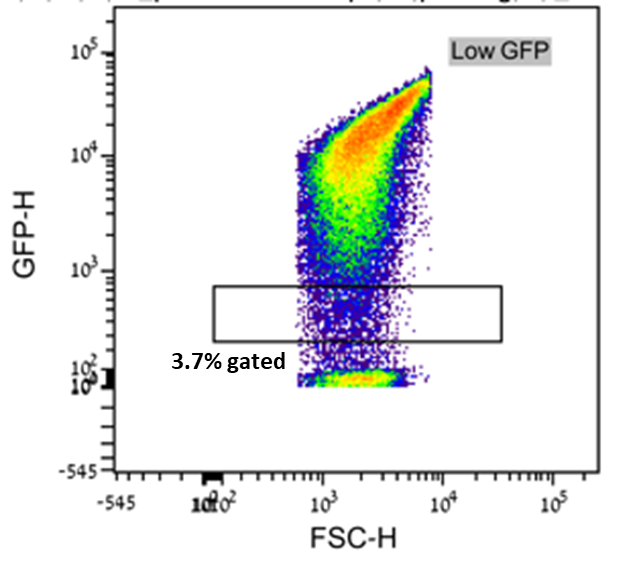

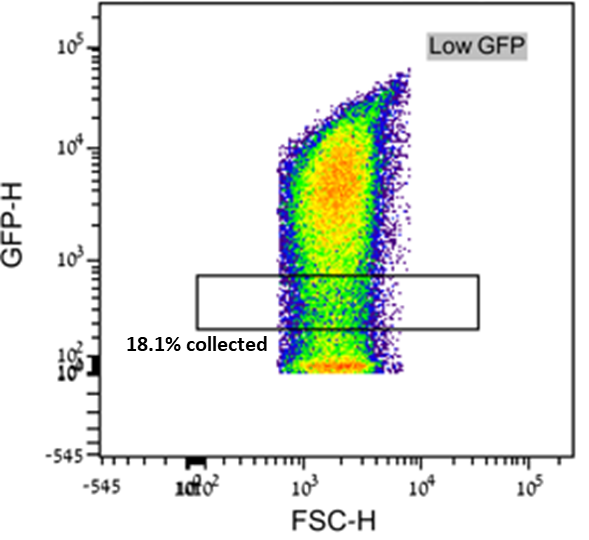


**Figure S4**

**A.**

**Clone 2**

**0 mg/L 6 mg/L**


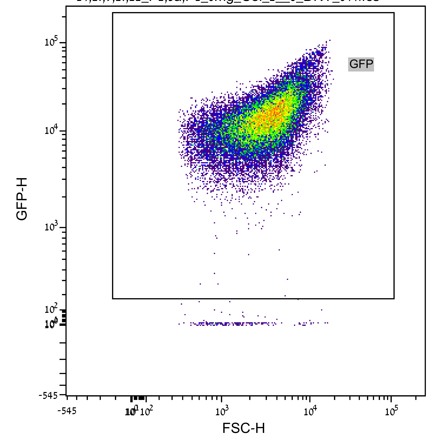
**
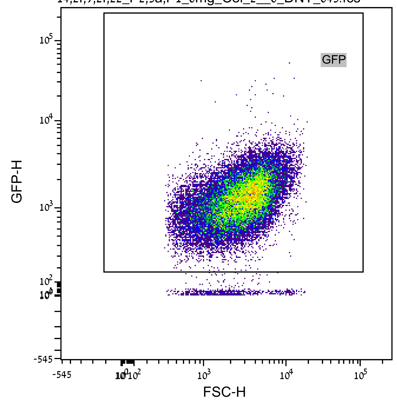
**

**Clone 5**

**0 mg/L 6 mg/L**


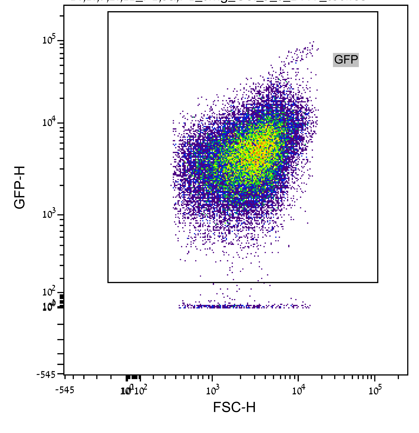

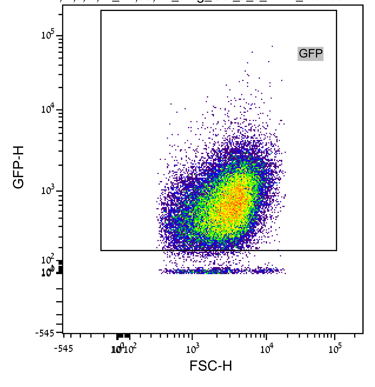


**Clone 9**

**0 mg/L 6 mg/L**
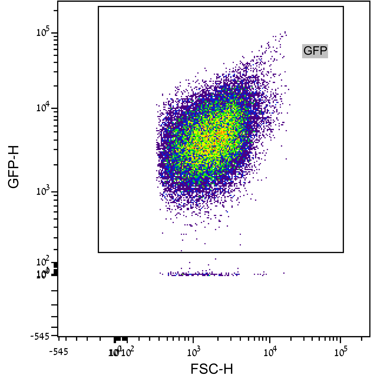

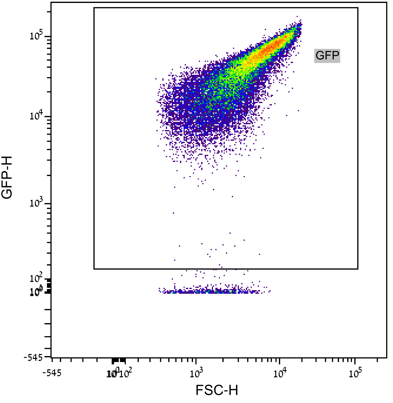


**B.**


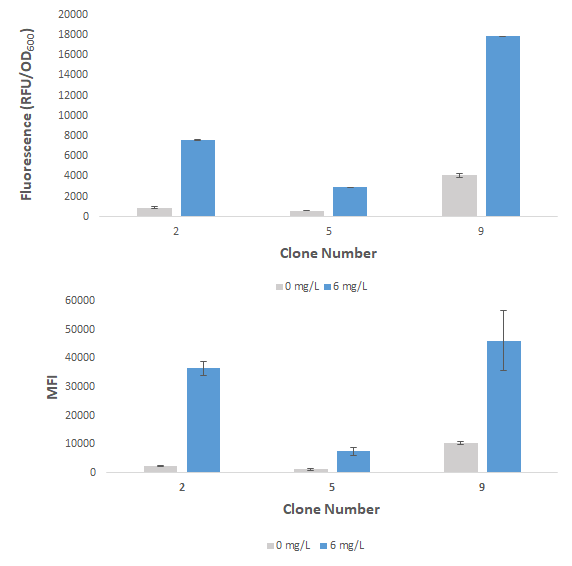


Figure S4. Comparable response to DNT in both FACS assay and plate assay. FACS density plot of clones 2, 5 and 9 induced by 6 mg/L DNT. Indicated clones were induced by either 0 mg/L or 6 mg/L DNT. The density plot of induced cells emitting GFP (GFP-H) vs forward scatter (FSC-H) MFI was calculated based on GFP positive cells. (i) Clone 2, MFI 1,362 vs 14,919 (ii) clone 5, MFI 901 vs 5,173 (iii) clone 9, MFI 5,183 vs 45,721 **(A).** Fluorescence response to  0mg/L  and 6 mg/L DNT. Top panel: Fluorescence response in 96 well plate. Bottom panel: MFI (mean fluorescence intensity units) in FACS assay  (**B**).

**Table S1.** List of primers

| **Primers** | Sequence 5’ 3’ |
| --- | --- |
| yhaJ_sall_FW | CCTGTCGACTACTAGAGCCTGC |
| 3prim_DbasI_Rv | GTTGGTCTCCAGCTTTTCTGGCAGCAATCGTTACGGAAAC |
|  |  |

**Table S2.** Amino acid and nucleotide sequence of *yhaJ* in pBS04 and in codon-optimized BS04_E

| **Vector** | **Amino Acid sequence** | **Nucleotide Sequence** |
| --- | --- | --- |
| pBS04 | MAKERALTLEALRVMDAIDRRGSFAAAADEMGRVPSALSYTMQKLEEELDVVLFDRSGHRTKFTNVGRMLLERGRVLLEAADKLTTDAEALARGWETHLTIVTEALVPTPAFFPLIDKLAAKANTQLAIITEVLAGAWERLEQGRADIVIAPDTHFRSSSEINSRKLYTLMNVYVAAPDHPIHQEPEPLSEVTRVKYRGIAVADTARERPVLTVQLLDKQPRLTVSTIEDKRQALLAGLGVATMPYPMVEKDIAEGRLRVVSPESTSEIDIIMVWRRDSMGEAKSWCLREIPKLFNGK | ATGGCCAAAGAAAGGGCATTAACGCTGGAAGCACTACGGGTTATGGATGCGATCGATCGCCGGGGCAGTTTTGCGGCGGCGGCGGATGAGATGGGACGCGTGCCTTCCGCACTTAGCTACACCATGCAAAAACTGGAAGAAGAGCTGGATGTGGTGCTGTTTGACCGCTCGGGCCATCGTACCAAATTCACCAATGTCGGGAGGATGTTGTTGGAGCGAGGGCGCGTTTTGCTGGAAGCCGCAGATAAACTGACTACTGATGCGGAAGCTCTCGCGCGCGGTTGGGAAACGCATCTCACCATTGTGACCGAAGCGCTGGTACCGACACCTGCCTTTTTCCCGTTAATCGACAAACTGGCGGCAAAAGCCAATACCCAACTGGCAATCATCACAGAAGTGCTGGCGGGGGCGTGGGAACGGCTGGAGCAGGGGCGGGCGGATATTGTTATCGCGCCGGATACGCATTTTCGTTCCTCGTCGGAGATCAACTCGCGCAAGCTCTATACGTTAATGAACGTCTACGTTGCCGCGCCTGATCACCCGATTCATCAGGAGCCGGAACCGTTATCTGAAGTGACGCGTGTGAAATATCGTGGAATTGCGGTGGCGGATACCGCTCGTGAGCGCCCGGTGTTGACCGTACAGCTGCTGGACAAACAGCCGCGCTTAACGGTGAGCACGATTGAAGATAAACGTCAGGCATTACTGGCGGGGCTTGGCGTGGCGACGATGCCGTATCCTATGGTCGAAAAAGATATTGCGGAAGGGCGGTTGCGTGTCGTCAGCCCGGAATCGACCAGCGAGATCGATATTATTATGGTCTGGCGTCGTGACAGTATGGGGGAAGCGAAATCCTGGTGTCTGCGGGAAATTCCCAAACTTTTTAACGGAAAATAA |
| pBS04_E |  | ATGGCTAAGGAACGTGCTCTTACCTTAGAGGCGCTTCGCGTAATGGACGCAATTGACAGAAGAGGATCGTTCGCAGCAGCTGCAGACGAAATGGGCCGAGTCCCGAGTGCTTTAAGTTATACAATGCAGAAGTTAGAGGAAGAATTAGACGTTGTTTTGTTCGATCGTAGTGGGCACCGGACAAAGTTTACTAACGTTGGTCGTATGCTGTTAGAACGTGGCCGTGTACTGTTGGAAGCAGCCGACAAGCTCACAACAGACGCAGAGGCACTGGCTCGTGGCTGGGAAACCCACTTAACAATAGTAACAGAGGCATTGGTTCCTACTCCAGCATTCTTTCCCCTGATAGATAAGCTTGCAGCCAAGGCGAACACACAGTTAGCTATTATTACAGAGGTTCTTGCCGGTGCTTGGGAGCGTTTAGAACAAGGTAGAGCCGACATAGTGATAGCTCCAGACACCCACTTCCGCTCTAGCTCCGAAATTAATAGTCGTAAATTATACACACTGATGAATGTTTATGTGGCTGCTCCAGACCATCCTATCCACCAAGAACCAGAGCCTCTGTCCGAGGTTACCCGCGTTAAGTACCGCGGCATCGCAGTAGCAGACACAGCAAGAGAAAGACCCGTCTTAACTGTGCAACTTCTTGATAAGCAACCTAGACTGACCGTTTCTACAATAGAGGACAAGAGACAAGCGCTGCTTGCCGGCCTCGGTGTTGCTACCATGCCTTACCCAATGGTGGAGAAGGACATAGCTGAGGGCCGATTACGGGTGGTGTCGCCTGAGAGTACATCAGAAATTGACATAATAATGGTATGGAGACGGGATTCTATGGGCGAGGCTAAGTCGTGGTGCTTACGAGAGATACCGAAGCTCTTCAACGGAAAGGAAACCTGA |

**Table S3 (link).** Top 150 enriched clones by NGS

<https://docs.google.com/spreadsheets/d/12WmyzZyJnKKUiUr9cdtaDwneVlwSlzMJ/edit?usp=sharing&ouid=104653599214773951741&rtpof=true&sd=true>

**Table S4 (link)**. DNT response statistics of clones tested post R3 FACS sorting. 34% of clones have a higher GFP when induced by DNT compared with the existing construct pBS04_G2 (G2a).

[https://docs.google.com/spreadsheets/d/12WmyzZyJnKKUiUr9cdtaDwneVlwSlzMJ/edit?usp=sharing&ouid=104653599214773951741&rtpof=true&sd=tru](https://docs.google.com/spreadsheets/d/12WmyzZyJnKKUiUr9cdtaDwneVlwSlzMJ/edit?usp=sharing&ouid=104653599214773951741&rtpof=true&sd=true)e

**Table** **S5**. Mutation heat map. All strain tested in E. coli BW25113 ΔygdD-ΔeutE harboring the mutated BS01a-based plasmids.

| **Clone** | **Positions mutated** | | | | | | **Also cloned in native codon usage** |
| --- | --- | --- | --- | --- | --- | --- | --- |
|  | **M31** | **D87** | **T154** | **I162** | **Q233** | **V274** |  |
| **2E** | F | E | N | M | E | T |  |
| **5E** | Q | D | N | V | V | V | **5N** |
| **10E** | F | N | N | Q | R | V | **10N** |
| **17E** | R | D | N | T | C | T | **17N** |
| **43E** | A | E | K | V | W | V | **43N** |
| **90E** | C | Y | N | C | I | V |  |
| **97E** | Y | T | K | C | Q | V |  |
| **135E** | L | S | K | I | I | T | **135N** |
| **39E** | F | D | N | K | F | V | **39N** |
| **119E** | C | D | N | M | k | T |  |
| **136E** | C | Q | N | L | V | T |  |

**Table S6.** Library mutation frequency analysis

| **Mutation position** | **31** | **87** | **154** | **162** | **233** | **274** |
| --- | --- | --- | --- | --- | --- | --- |
| ***** | 0.02% | 0.05% | 0.11% | 0.03% | 0.29% | 0.07% |
| **A** | 3.11% | 7.91% | 4.52% | 4.62% | 3.27% | 4.99% |
| **C** | 2.62% | 4.37% | 3.93% | 4.53% | 3.89% | 4.03% |
| **D** | 2.54% | 28.41% | 3.09% | 3.82% | 3.25% | 4.48% |
| **E** | 2.47% | 4.14% | 3.25% | 3.01% | 3.41% | 3.29% |
| **F** | 3.04% | 3.43% | 4.16% | 4.05% | 2.77% | 2.85% |
| **G** | 2.43% | 2.88% | 3.50% | 3.18% | 3.66% | 2.99% |
| **H** | 3.05% | 3.84% | 4.79% | 4.03% | 4.51% | 4.12% |
| **I** | 5.30% | 3.28% | 4.13% | 27.54% | 2.99% | 3.49% |
| **K** | 4.90% | 3.20% | 3.19% | 3.77% | 3.23% | 3.80% |
| **L** | 8.01% | 3.93% | 3.94% | 3.15% | 5.27% | 4.37% |
| **M** | 28.94% | 3.25% | 3.42% | 4.46% | 3.66% | 3.69% |
| **N** | 4.09% | 4.17% | 3.91% | 4.57% | 2.42% | 3.12% |
| **P** | 6.07% | 3.57% | 3.29% | 5.20% | 0.37% | 4.55% |
| **Q** | 5.38% | 3.35% | 2.62% | 2.91% | 28.84% | 4.92% |
| **R** | 3.38% | 3.25% | 3.69% | 2.59% | 5.35% | 3.91% |
| **S** | 3.45% | 3.89% | 4.24% | 4.04% | 5.11% | 3.44% |
| **T** | 4.38% | 3.54% | 27.76% | 4.38% | 4.24% | 3.64% |
| **V** | 3.86% | 2.38% | 4.51% | 4.04% | 4.38% | 27.13% |
| **W** | 0.33% | 3.65% | 4.28% | 2.55% | 4.62% | 3.39% |
| **Y** | 2.64% | 3.50% | 3.65% | 3.51% | 4.48% | 3.69% |
